# Engineered Ureolytic Microorganisms Can Tailor the Morphology and Nanomechanical Properties of Microbial-Precipitated Calcium Carbonate

**DOI:** 10.1038/s41598-019-51133-9

**Published:** 2019-10-11

**Authors:** Chelsea M. Heveran, Liya Liang, Aparna Nagarajan, Mija H. Hubler, Ryan Gill, Jeffrey C. Cameron, Sherri M. Cook, Wil V. Srubar

**Affiliations:** 1 Department of Civil, Environmental, and Architectural Engineering, University of Colorado Boulder, ECOT 441 UCB 428, Boulder, Colorado 80309-0428 USA; 2 Renewable and Sustainable Energy Institute, University of Colorado Boulder, 027 UCB Suite N321, Boulder, Colorado 80309 USA; 3 Department of Chemical and Biological Engineering, University of Colorado Boulder, 596 UCB, Boulder, Colorado 80309 USA; 4 Department of Biochemistry, University of Colorado Boulder, 596 UCB, Boulder, Colorado 80309 USA; 5 National Renewable Energy Laboratory, 15013 Denver West Parkway, Golden, Colorado 80401 USA; 6 Materials Science and Engineering Program, 027 UCB, Boulder, Colorado 80303 USA

**Keywords:** Microbiology, Engineering

## Abstract

We demonstrate for the first time that the morphology and nanomechanical properties of calcium carbonate (CaCO_3_) can be tailored by modulating the precipitation kinetics of ureolytic microorganisms through genetic engineering. Many engineering applications employ microorganisms to produce CaCO_3_. However, control over bacterial calcite morphology and material properties has not been demonstrated. We hypothesized that microorganisms genetically engineered for low urease activity would achieve larger calcite crystals with higher moduli. We compared precipitation kinetics, morphology, and nanomechanical properties for biogenic CaCO_3_ produced by two *Escherichia coli* (*E. coli*) strains that were engineered to display either high or low urease activity and the native producer *Sporosarcina pasteurii*. While all three microorganisms produced calcite, lower urease activity was associated with both slower initial calcium depletion rate and increased average calcite crystal size. Both calcite crystal size and nanoindentation moduli were also significantly higher for the low-urease activity *E. coli* compared with the high-urease activity *E. coli*. The relative resistance to inelastic deformation, measured *via* the ratio of nanoindentation hardness to modulus, was similar across microorganisms. These findings may enable design of novel advanced engineering materials where modulus is tailored to the application while resistance to irreversible deformation is not compromised.

## Introduction

Microbially induced calcium carbonate (CaCO_3_) precipitation (MICP) is ubiquitous in nature and is responsible for CaCO_3_ formations in terrestrial and marine environments^1–3^. MICP has been widely used for a variety of applications, including soil stabilization^4^, *in situ* cement repair^5,6^, oil and gas well fracture-sealing^7^, bioremediation of metals^8,9^, and sealing subsurface fractures to mitigate leakage from geologically sequestered CO_2_^10^. Biogenic CaCO_3_ mineralization is instigated by changes to solution chemistry local to microorganisms^4,5^. Microorganisms such as the soil bacterium *Sporosarcina pasteurii* (*S. pasteurii*, previously known as *Bacillus pasteurii*) produce the enzyme urease. This enzyme hydrolyzes urea to form ammonia and carbamic acid, which then spontaneously hydrolyzes to ammonia and carbonic acid. Near the bacterial cell, pH increases with the generation of hydroxide ions, and shifts solution equilibria towards the availability of bicarbonate and carbonate ions. When calcium (Ca^2+^) ions are available, CaCO_3_ is formed^5,11,12^. The negatively-charged bacterial surface often serves as a nucleation center for CaCO_3_ precipitation, leading to the formation of crystals with bacterial imprints^11^.

Tailoring the morphology and material properties of biogenic CaCO_3_ could confer new functionalities to biocement and would make possible novel advanced engineering applications, such as hybrid living building materials and functionally-graded composites and films. Achieving these applications would require the precipitation of stable CaCO_3_ crystals with tailored moduli and high hardness. Different CaCO_3_ polymorphs have a range of stabilities. Calcite is the most stable polymorph, while aragonite is relatively less stable and vaterite is only metastable. Crystal size also affects stability. Larger calcite crystals are generally more stable and are often rounded when precipitated in the presence of microorganisms^13–15^. Round crystal morphology would be expected to improve the rheological properties of biocement^16^. Higher or lower microscale modulus of CaCO_3_ crystals is not universally more desirable. Instead, tailoring the modulus of CaCO_3_ would facilitate creating functionally-graded interfaces and modulating the structural stiffness of biogenic composites. On the other hand, high hardness is generally valuable for the desired applications. Nacre, brachiopods, otoliths, bone, and enamel are all dominantly composed of strength-bearing brittle phases (such as CaCO_3_ or calcium phosphate minerals), yet gain toughness and resilience from the presence of a small quantity of relatively ductile polymer^17–20^. These biogenic materials have up to 100% higher hardness relative to their modulus compared to inorganic counterparts, such as flask-precipitated CaCO_3_^20^.

Previous efforts with *S. pasteurii* and other wild-type microorganisms have changed the solution saturation state to affect some degree of control over CaCO_3_ morphology and material properties. Higher saturation states, achieved with choice of media and ureolytic microorganism, can encourage the formation of vaterite^5,15,21^. These crystals are larger and less stiff than calcite, but are metastable and tend to transform to aragonite or calcite^15,21–23^. The ability to engineer advanced functional biocements and composites would be improved if calcite, as opposed to vaterite, was precipitated with desirable range of morphologies and material properties. However, control over native ureolytic microorganisms and their mineral precipitates is limited. *S. pasteurii* has been UV-mutated to introduce variance in urease expression^9^, but more controlled genetic engineering is challenging because the genomes of these microorganisms are neither fully understood nor easily tractable with synthetic biology tools.

Genetically engineered ureolytic *Escherichia coli (E. coli)* may accomplish greater control over the morphology and material properties of biogenic calcite. While *S. pasteurii* and other native ureolytic microorganisms are difficult to engineer, the genome of *E. coli* is well-understood and can be readily genetically modified to express urease with different activities. We previously demonstrated that engineered ureolytic *E. coli* produces CaCO_3,_ and constructed several strains with a range of urease activities and corresponding precipitation kinetics^24^. Our engineered strain *E. coli* HB101/ure-integration, with relatively low urease activity and slow precipitation kinetics, produced larger calcite crystals than other *E. coli* strains with greater urease activity, such as *E. coli* HB101/pBU11. Changing CaCO_3_ precipitation kinetics also has the potential to affect crystal material properties. For instance, previous work demonstrated that modulus can be increased by slowing crystal growth, which minimized crystal defects and lattice strain^25^. Unlike changing the solution conditions, which confounds the effect of the microorganism with solution chemistry, the use of genetically engineered *E. coli* with higher or lower urease activity allows testing the specific role of precipitation kinetics on crystal morphology and material properties.

The purposes of this study were (1) to quantify and compare the precipitation kinetics for *E. coli* that were engineered to have different urease activities and (2) to ascertain the influence of precipitation kinetics on CaCO_3_ morphology and material properties (*i.e*., microscale modulus and hardness). We hypothesized that the ability to tailor the morphology and material properties of biogenic CaCO_3_ would be improved by genetically controlling the kinetics of biogenic crystal precipitation, such that an engineered *E. coli* strain with relatively slower CaCO_3_ precipitation kinetics would produce larger crystals with higher nanoindentation moduli.

## Results

### Microorganism viability, urease activity, and solution chemistry during the 7-day experiment

**Figure 1:**
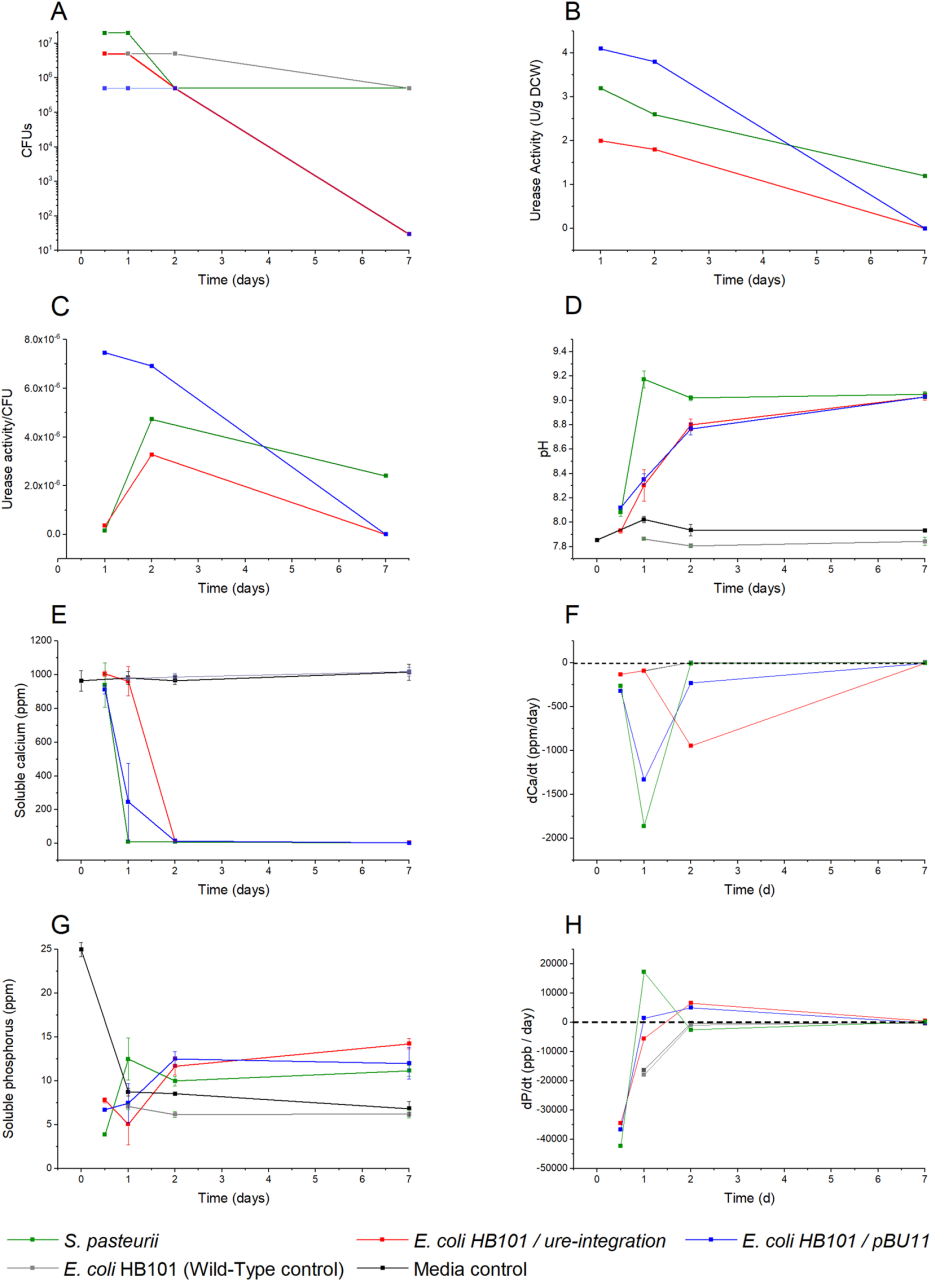
Cell growth and precipitation kinetics throughout 7d. (**A**) Colony forming units were measured for microorganisms. All strains retained viability throughout the 7d experiment. (**B**) Urease activity declined across the 7d experiment for all microorganisms. (**C**) Urease activity normalized to CFUs was at a maximum at 1d for *E. coli* HB101/pBU11 and 2d for the other two ureolytic microorganisms (**D**) Native and engineered ureolytic microorganisms raised the solution pH, although at different rates. (**E**) Soluble Ca was depleted at different times for ureolytic strains; the slowest total Ca depletion was observed for the engineered strain with the lowest urease activity, *E. coli* HB101/ure-integration. Ca was slightly lowered in controls with the precipitation of CaP. (**F**) The rate of calcium depletion, dCa/dt, differed by microorganism. The dCa/dt at beginning of calcite nucleation (12h for *S. pasteurii* and *E. coli* HB101/pBU11, 1d for *E. coli* HB101/ure-integration) was related to the urease activity of the microorganism and inversely related to calcite crystal size at the study endpoint. (**G**) Soluble P initially decreased for ureolytic strains and control conditions with the spontaneous precipitation of CaP. (**H**) The rate of P depletion in solution, dP/dt, varied with microorganism such that phosphorus was initially depleted and then returned to solution with calcite nucleation.

We monitored the viability and urease activity for two engineered ureolytic *E. coli* strains, *E. coli* HB101/pBU11 and *E. coli* HB101/ure-integration, and the native producer *S. pasteurii*. Over 7 days, colony forming units (CFUs) (Fig. 1A) and urease activity (Fig. 1B) decreased for all three ureolytic microorganisms. However, urease activity normalized to CFU (Fig. 1C) decreased with time for *E. coli* HB101/pBU11 but reached a maximum at 2d for the other two ureolytic microorganisms. Only *S. pasteurii* had measurable urease activity at 7d.

We then quantified the precipitation kinetics of each microorganism through measuring pH, calcium (Ca), and phosphorus (P) at each timepoint. Each of the three ureolytic microorganisms raised the solution pH compared to Media (no cell) and Wild-Type (*E. coli* HB101 with no urease genes) controls over the course of the 7d experiment (Fig. 1D). Soluble Ca measured from inductively coupled optical emission spectroscopy (ICP-OES) initially decreased in all cultures, including the two controls. However, as expected, Ca was most reduced for solutions containing ureolytic microorganisms (Fig. 1E), with most of the Ca in solution depleted by day 2. The rate of Ca depletion (dCa/dt) varied by microorganism (Fig. 1F). Through 1d, dCa/dt was not different between *E. coli* HB101/ure-integration and controls. At 2d, dCa/dt was at a maximum for the low-urease activity engineered microorganism. For the higher-urease activity engineered microorganism, *E. coli* HB101/pBU11, as well as the native producer *S. pasteurii*, dCa/dt was greatest at 1d and then approached zero at 2–7d. At the timepoints most closely representing calcite nucleation as verified by XRD and SEM (12h for *S. pasteurii* and *E. coli* HB101/pBU11; 1d for *E. coli* HB101/ure-integration), dCa/dt increased in the order of measured urease activity. Specifically, dCa/dt was lowest for *E. coli* HB101/ure-integration, followed by *S. pasteurii*, and then *E. coli* HB101/pBU11.

Soluble P concentration varied considerably throughout the week-long experiment (Fig. 1G). The rate of P depletion (dP/dt) was initially large for all three ureolytic microorganisms and later reversed as P returned to solution, indicating dissolution of P-containing precipitates (Fig. 1H). As with dCa/dt, the timing of this transition depended on microorganism. For the low-urease activity microorganism, P returned to solution at days 2–7, whereas P dissolution occurred a day earlier for the two more ureolytic microorganisms. Finally, consistent with SEM and XRD observation of calcium phosphate crystals, the wild-type and media controls demonstrated high initial dP/dt that then tapered towards zero. Unlike with ureolytic microorganisms, the control conditions did not return P to solution during the 7d experiment.

### Mineral phases assessed by XRD

**Figure 2:**
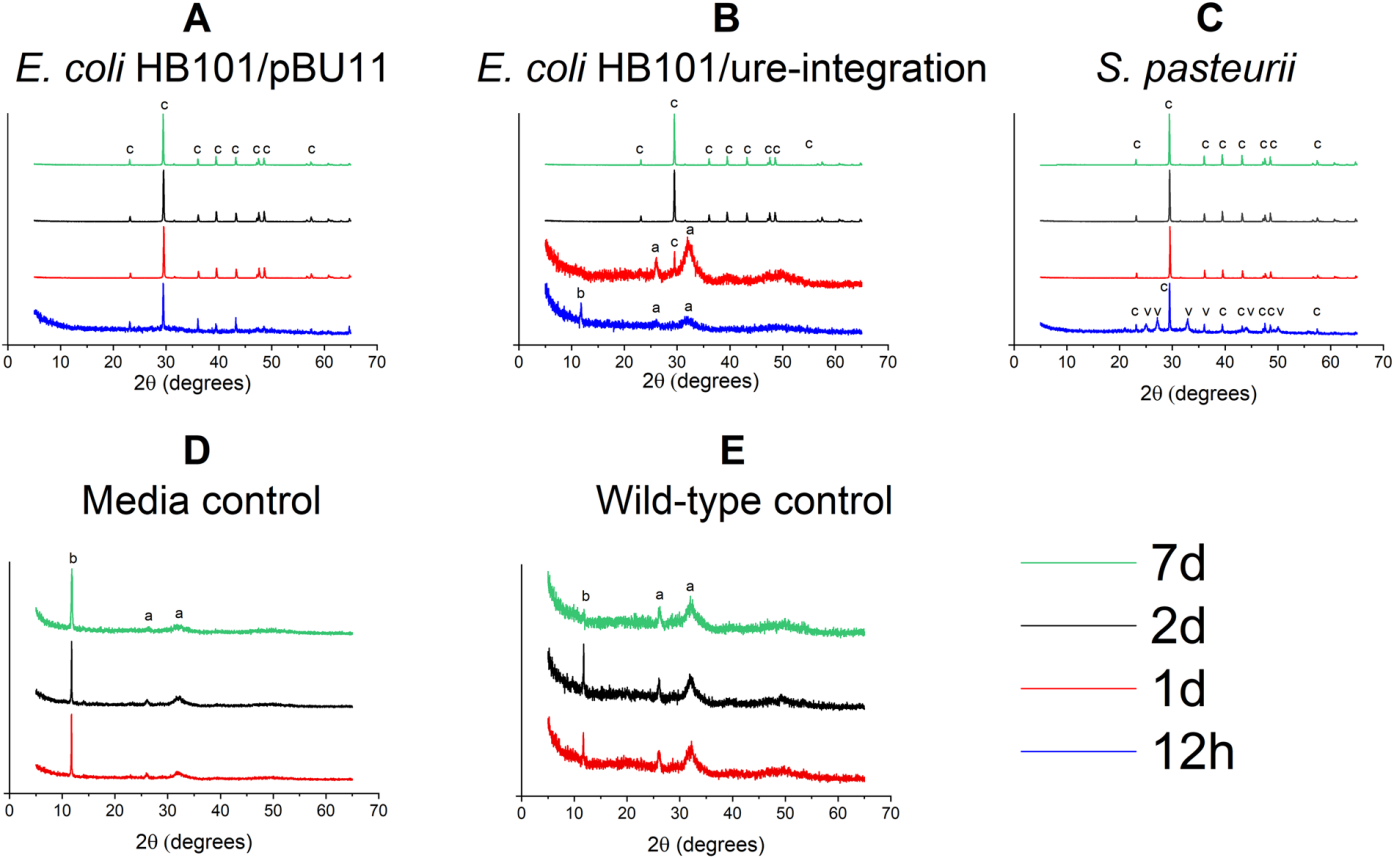
XRD detection of CaCO_3_ and CaP precipitated by ureolytic and control cultures throughout 7d. (**A**) *E. coli* HB101/pBU11 precipitated only calcite at all time points. (**B**) *E. coli* HB101/ure-integration initially precipitated brushite and apatite. At 1d, the dominant calcite peak emerged. At 2–7d, only calcite was detected. (**C**) *S. pasteurii* precipitated vaterite (v) and calcite (c) at 12h, and only calcite at later timepoints. (**D**) For all time points, the Media Control culture precipitated calcium phosphate phases, including brushite (b) and apatite (a). No CaCO_3_ was detected. (**E**) The Wild-Type Control precipitated calcium phosphate phases, but not CaCO_3._

We employed x-ray diffraction (XRD) to characterize which phases of CaCO_3_ and calcium phosphate (CaP) were precipitated by each microorganism across the 7d experiment. XRD revealed that Media and Wild-Type controls produced CaP, namely brushite and apatite (Fig. 2D,E). The urea-CaCl_2_ growth media contains P in the ingredient Difco (‘nutrient broth’). No CaCO_3_ was identified for the controls at any time point. For *S. pasteurii*, only CaCO_3_ was detected across the course of the experiment, although CaCO_3_ polymorphs varied with time. For the *E. coli* HB101/pBU11 strain, only calcite was detected at each time point (Fig. 2A). *E. coli* HB101/ure-integration produced calcite and CaP at 12h and 1d, and only calcite for 2d–7d (Fig. 2B). At 12h, *S. pasteurii* produced vaterite and calcite, while only calcite was detected for 1d–7d (Fig. 2C).

The XRD results demonstrated good correspondence with the changes in solution chemistry noted over the 7d experiment. Control conditions did not raise pH and only produced CaP. Changes to pH and soluble Ca were evident as early as 12h for *S. pasteurii* and *E. coli* HB101/pBU11, which aligned with the detection of prominent CaCO_3_ peaks at 12h onwards. By contrast, for the lower urease-activity *E*. *coli* HB101/ure-integration, neither pH nor soluble Ca were appreciably changed until 2d. Calcite and CaP were detected during this first 2d. From 2–7d, when pH was elevated by *E. coli* HB101/ure-integration to similar levels as achieved the other two microorganisms, XRD only detected calcite.

### Crystal morphology

**Figure 3:**
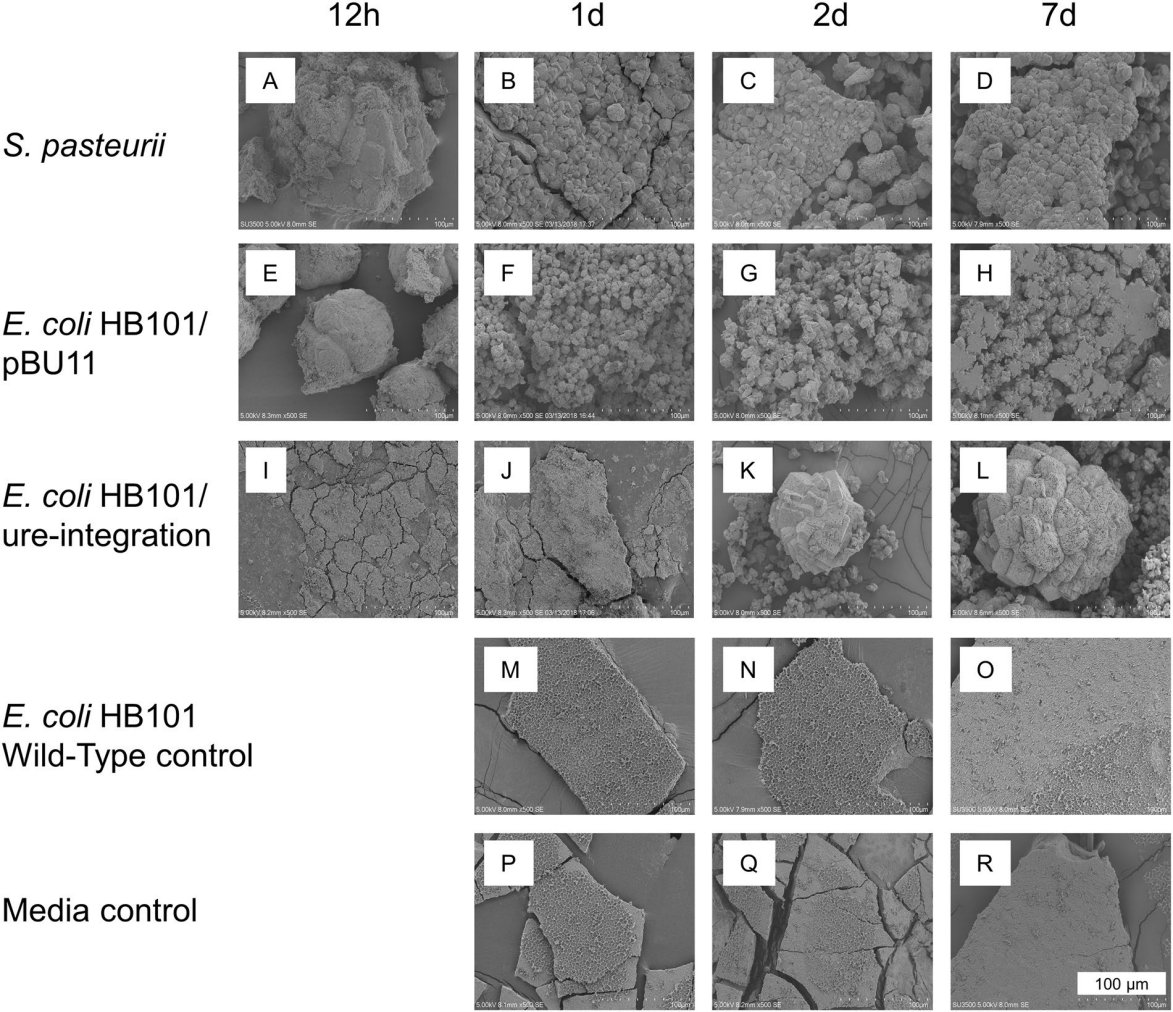
Longitudinal evolution of CaCO_3_ morphology across 7d for engineered *E. coli* and control comparisons. *S. pasteurii* (**A–D**) and *E. coli* HB101/pBU11 (**E–H**) both had an evolution over time from large, poorly faceted structures at 12h to smaller, faceted polycrystalline structures with abundant bacterial casting at 1–7d. *E. coli* HB101/ure-integration (**I–L**) initially formed calcium phosphate nanocrystals until 2d, when large, round, highly casted CaCO_3_ crystal structures appeared. At 7d, the average calcite crystal size was smallest for *E. coli* HB101/pBU11, followed by *S. pasteurii* and then *E. coli* HB101/ure-integration. Wild-Type (**M–O**) and Media (**P–R**) controls produced only calcium phosphate nanocrystalline sheets which did not vary in morphology from 1–7d. All SEM images shown at 500×.

To evaluate the hypothesis that precipitation kinetics influence crystal morphology, we assessed crystal size and shape using scanning electron microscopy (SEM) at all time points. *S. pasteurii* produced large (~100 µm) structures at 12h with regions of well crystallized material with bacterial casts alongside neighboring regions of disorganized and uncasted material (Fig. 3A–D). By 1d, and continuing through 7d, crystals precipitated by *S. pasteurii* recrystallized to smaller and more uniform crystalline structures with bacterial casts. The crystals precipitated by *E. coli* HB101/pBU11 demonstrated a similar transition from large, mixed structures at 12h to smaller calcites with clear bacterial casts (Fig. 3E–H). *E. coli* HB101/ure-integration produced nanocrystalline sheets at 12h and 1d (Fig. 3I–L). By 2d, and continuing through 7d, precipitates from *E. coli* HB101/ure-integration were well-crystallized, and casted calcites and were often very large. From measurement of crystal sizes at 7d, the average size of calcite crystals produced by *E. coli* HB101/ure-integration was larger than for crystals precipitated by *S. pasteurii* (+371%, p < 0.001) and much larger than for crystals precipitated by *E. coli* HB101/pBU11 (+1924%, p < 0.001) (Table 1). SEM images revealed that Media and Wild-Type controls produced nanocrystalline sheets that did not vary in morphology from 1–7d (Fig. 3M–R). The morphologies observed in the control conditions were similar to those from the earliest timepoints (12h–1d) for *E. coli* HB101/ure-integration.

**Table 1:** Morphology and nanomechanical properties of biocalcite crystals.

	*S. pasteurii*	*E. coli* HB101/pBU11	*E. coli* HB101/ure-integration
Crystal area (µm^2^) Microorganism: p < 0.001	72.1 ± 11.3	16.8 ± 1.70	340 ± 21.5*^#^ *p < 0.001 ^#^p < 0.001
Indentation modulus (E_i_, GPa) Microorganism: p = 0.031	36.4 ± 1.49	33.7 ± 0.541	38.7 ± 1.02^#^ p = 0.025
Hardness (H_i_, GPa) Microorganism: p > 0.05	2.62 ± 0.0516	2.51 ± 0.0823	2.66 ± 0.0599
H_i_/E_i_ Microorganism: p > 0.05	7.50E-2 ± 2.15E-3	7.83E-2 ± 2.63E-3	7.22E-2 ± 1.85E-3
H (GPa) Microorganism: p > 0.05	4.37 ± 0.0872	4.31 ± 0.176	4.46 ± 0.124

Data are presented as mean +/− standard error of the mean. One-way ANOVA result for the main effect of microorganism is reported in the left column. *Indicates significant difference compared with *S. pasteurii*; ^#^Indicates significant difference compared with *E. coli* HB101/pBU11. Post-hoc tests were adjusted for family-wise error with the Tukey procedure.

### Chemical composition of minerals via SEM-EDS and WDS

**Figure 4:**
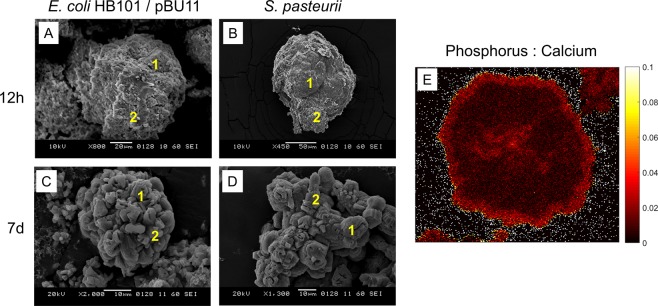
Phosphorus participates in biogenic CaCO_3_ crystal nucleation and growth. SEM-EDS at 12h for (**A**) *E. coli* HB101/pBU11 and (**B**) *S. pasteurii* show well-defined, casted regions of CaCO_3_ (1) adjacent to poorly organized CaP (2) on the same structure. By 7d, (**C**) *E. coli* HB101/pBU11 and (**D**) *S. pasteurii* both show only CaCO_3_. (**E**) WDS revealed that P is present at the CaCO_3_ crystal core as well as in an outer surface ring. This pattern of P distribution was observed for all three microorganisms.

Because XRD revealed the presence of CaP phases for controls as well as for ureolytic microorganisms at early time points, we employed SEM-EDS to identify CaP in study precipitates. SEM-EDS confirmed that nanocrystalline sheets produced by Media and Wild-Type controls contained both Ca and P. For biogenic precipitates from *S. pasteurii* and *E. coli* HB101/pBU11 collected at 12h, the faceted and casted regions of these precipitates were identified as CaCO_3_. Meanwhile, disorganized regions of the same structures were identified as CaP (Fig. 4A,B). At later timepoints, only CaCO_3_ was identified in crystals precipitated by *S. pasteurii* and *E. coli* HB101/pBU11 (Fig. 4C,D). By contrast, *E. coli* HB101/ure-integration produced mostly CaP until day 2, at which point all crystals studied with SEM-EDS were composed of only CaCO_3_. We also sought to identify where P was present in the interior of biogenic CaCO_3_ at the study endpoint. Cross-sections of crystals precipitated by each of the three ureolytic microorganisms were mapped using wavelength dispersive spectroscopy (WDS). Crystals from all three ureolytic microorganisms contained substantial P content (P:Ca ~ 5–10%) in the core of the crystal, as well as within a ring at the crystal surface (Fig. 4E).

### Mechanical properties via nanoindentation

**Figure 5:**
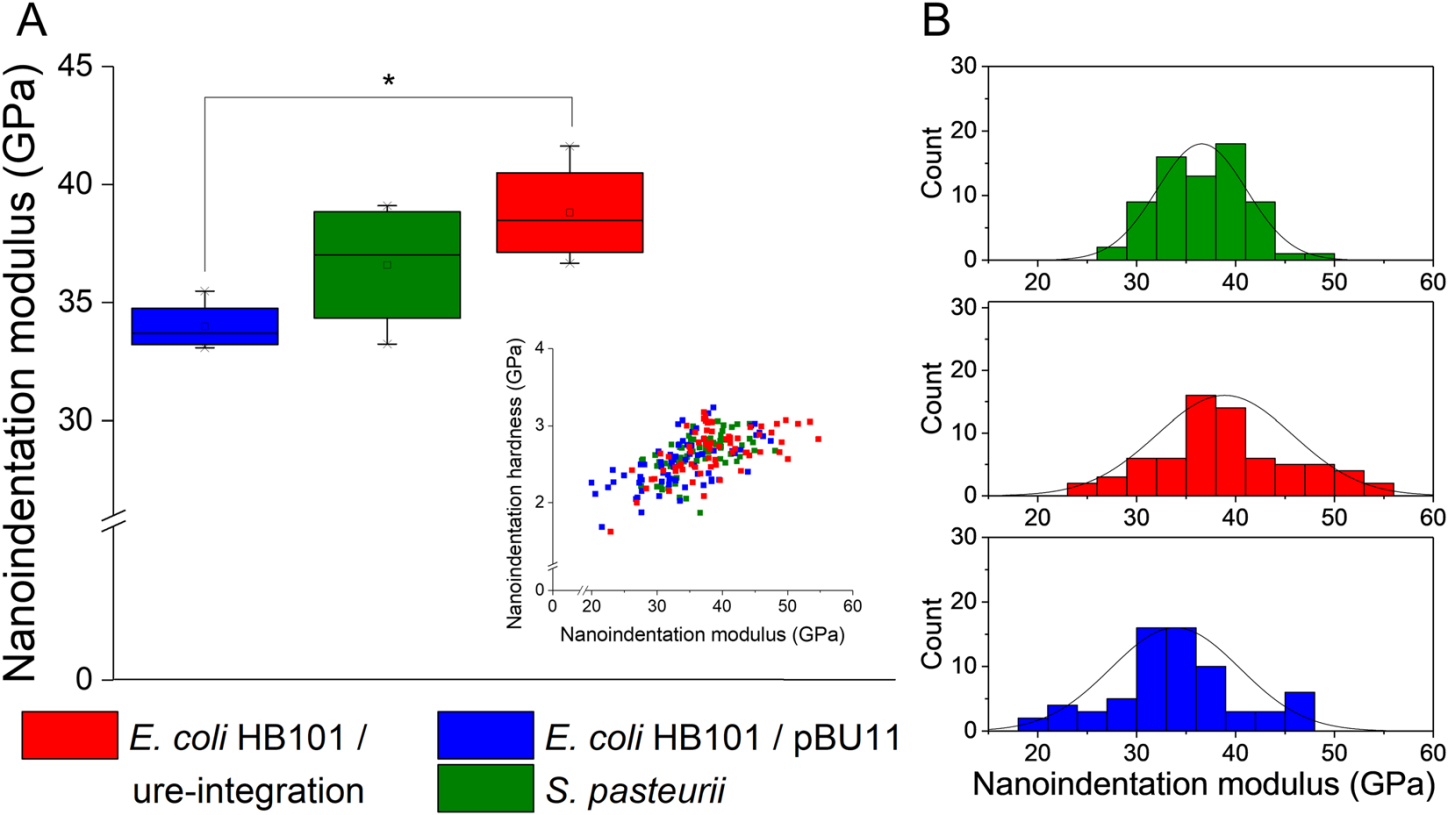
Nanomechanics of biogenic calcite can be tailored with engineered *E. coli*. (**A**) E_i_ was significantly higher for the engineered *E. coli* HB101/ure-integration (lower urease activity) compared with *E. coli* HB101/pBU11 (higher urease activity). Inset: the ratio of H_i_ to E_i_ does not change with precipitating microorganism, demonstrating that resistance to permanent deformation is not compromised with choice of higher or lower E_i_. (**B**) Compared with *E. coli* HB101/pBU11 and *S. pasteurii*, distributions of moduli for *E. coli* HB101/ure-integration were shifted towards higher values.

We utilized nanoindentation to evaluate whether microscale mechanical properties of biogenic CaCO_3_ depend on the precipitating microorganism. From ANOVA, there was a main effect of microorganism (p = 0.031) on mean nanoindentation modulus, E_i_. Tukey post-hoc testing revealed that the *E. coli* HB101/ure-integration strain produced calcite crystals with significantly higher moduli (E_i_, +14.8%, p = 0.025) than *E. coli* HB101/pBU11 (Fig. 5A). Calcites from *S. pasteurii* exhibited moduli that were not statistically different from either engineered *E. coli* strain.

Histograms of raw (*i.e*., not averaged) nanoindentation moduli for each microorganism showed that all ureolytic microorganisms produced calcites with a range of E_i_, yet *E. coli* HB101/ure-integration produced stiffer crystals with greater frequency (Fig. 5B). While 34.8% of indents from crystals from *E. coli* HB101/ure-integration had E_i_ of at least 40 GPa, this percentage declined to 22.1% and 14.7% for crystals precipitated by *S. pasteurii* and for *E. coli* HB101/pBU11, respectively. Average crystal size and average nanoindentation modulus, measured for crystals collected from the same cultures, were found to significantly correlate (p = + 0.830, p < 0.05).

Neither indentation hardness, H_i_, nor hardness, H (as determined from Eq. 5), significantly differed with ureolytic microorganism (Table 1). The ratio of H_i_/E_i_, interpreted as the relative material resistance to inelastic deformation during nanoindentation, were similar for all three ureolytic microorganisms (Fig. 5A inset**)**. Thus, although E_i_ depended on which microorganism precipitated the crystal, the relative resistance to inelastic deformation was similar for all crystals across all ureolytic microorganisms.

### Assessment of crystal phase and composition at indentation sites

We aimed to understand why the low-urease activity engineered strain (*E. coli* HB101/ure-integration) produced significantly higher modulus CaCO_3_ crystals than the low-urease activity engineered strain (*E. coli* HB101/pBU11). A Raman spectrum collected at the same location as each indent was used (1) to identify the phase(s) of CaCO_3_ present, (2) to measure the relative size and perfection of the crystal, and (3) to identify the presence of one or more CaP phases. Of note, though CaP was not identified in bulk 7d samples using XRD, Raman spectroscopy can detect trace phases below the XRD detection limit at microscale observation sites.

All Raman spectra contained calcite peaks, while no additional polymorphs of CaCO_3_ (*i.e*., vaterite or aragonite) were identified, indicating that differences in nanoindentation properties were not due to CaCO_3_ phase. Weak CaP peaks were detected for some indent locations for each of the three ureolytic microorganisms, revealing the presence of minor quantities of CaP. No differences in mean crystal size (A280/A712) or perfection (FWHM of *v*_1_ carbonate) or CaP phases (brushite:*v*_1_ carbonate, apatite:*v*_1_ carbonate, carbonated apatite:*v*_1_ carbonate) of precipitates were found between microorganisms (Table 2). However, relationships between CaP content and E_i_ differed by microorganism. For crystals precipitated by *E. coli* HB101/pBU11, for example, reduced E_i_ was significantly correlated with greater apatite:*v*_1_ carbonate (r^2^ = 0.25, p = 0.001) and brushite:*v*_1_ carbonate (r^2^ = 10.2%, p = 0.042). These measures demonstrated a high degree of intercorrelation (r^2^ = 0.514) and thus cannot be interpreted separately. Instead, these findings show that E_i_ is reduced for higher amounts of CaP phases at indentation sites on precipitates produced by *E. coli* HB101/pBU11. By contrast, for crystals precipitated by *E. coli* HB101/ure-integration and *S. pasteurii*, the presence of CaP was not significantly correlated with reduced E_i_.

**Table 2:** Measures from Raman spectroscopy for biocalcite crystals.

	*S. pasteurii*	*E. coli* HB101/pBU11	*E. coli* HB101/ure-integration
Apatite: *v*_1_ carbonate Microorganism: p > 0.05	2.44E-3 ± 0.501E-3	4.64E-3 ± 2.23E-3	4.96E-3 ± 2.50E-3
Brushite: *v*_1_ carbonate Microorganism: p > 0.05	6.80E-3 ± 5.95E-3	13.6E-3 ± 13.5E-3	26.9E-3 ± 9.54E-3
Carbonated apatite: *v*_1_ carbonate Microorganism: p > 0.05	3.36E-2 ± 1.6E-2	9.29E-2 ± 4.07E-2	2.61E-2 ± 1.16E-2
A280/A712 Microorganism: p > 0.05	4.88 ± 0.550	4.73 ± 1.63	5.25 ± 1.57
FWHM of *v*_1_ carbonate Microorganism: p > 0.05	4.47 ± 0.0827	4.40 ± 0.0758	4.39 ± 0.122

Data are presented as mean +/− standard error of the mean. One-way ANOVA result for the main effect of microorganism is reported in the left column.

## Discussion

The purpose of this study was to determine whether morphology and material properties of biogenic CaCO_3_ could be controlled by engineering ureolytic microorganisms with different urease activities and, consequently, precipitation kinetics. We studied biogenic crystal precipitates from three ureolytic microorganisms: the native ureolytic *S. pasteurii*, the engineered *E. coli* HB101/pBU11 with similar urease activity as *S. pasteurii*, and the engineered *E. coli* HB101/ure-integration strain with lower urease activity than either of the other two microorganisms. To understand how nucleation, growth, and stability of crystal precipitates differed for these three microorganisms, the solution chemistry, crystal phase, and crystal morphology were compared at multiple time points from 12h through 7d and compared with control conditions. At 7d, microscale chemistry and elemental analysis of biogenic CaCO_3_ from the three microorganisms were performed with Raman spectroscopy and WDS, respectively, while microscale mechanical properties were assessed using nanoindentation.

All three microorganisms produced only calcite and no other polymorphs of CaCO_3_ by the end of the 7d experiment. Vaterite was observed as a transient phase at 12h for crystals precipitated by *S. pasteurii*, while no metastable phases of CaCO_3_ were observed at any timepoint for either engineered *E. coli* strain. Importantly, metastable phases may have been present for engineered *E. coli* strains before 12h, or in sub-detectable quantities. Alternatively, vaterite precipitated by *S. pasteurii* may have been stabilized by extracellular polymeric substances (EPS), as has been witnessed in prior work^1,14^. Unlike *S. pasteurii*, *E. coli* HB101 does not produce biofilm^26,27^. Ultimately, the transient vaterite may not have great consequence to the mature calcite crystals, as all detectable vaterite transformed into calcite during the experimental timeframe.

The kinetics of mineralization and resulting crystal morphology depended on microorganism ureolytic activity. The initial rate of calcium depletion from solution (*i.e*., the rate of calcium depletion most closely associated with calcite nucleation for each microorganism) increased with the urease activity of the precipitating microorganism. The low-urease activity microorganism (*E. coli* HB101/ure-integration) had the lowest initial calcium depletion rate and the high-urease activity microorganism (*E. coli* HB101/pBU11) had the highest initial calcium depletion rate. The average size of crystals measured at 7d was also inversely related to both the urease activity and the initial calcium depletion rate. At the study endpoint, the average crystal size for the low-urease engineered microorganism was almost 20 times larger than for the high-urease engineered microorganism. The growth of cells is another important consideration for crystal nucleation and growth, as cells produce urease and can also serve as nucleation centers. *S. pasteurii* maintained higher viability than the two engineered *E. coli* strains throughout the study. This may have influenced why *S. pasteurii* most quickly depleted total Ca in solution. However, in our study, neither initial calcium depletion rate nor crystal size were clearly related to differences in colony formation units at any point of the experiment.

Our findings that lower urease activity microorganisms produced slower initial nucleation kinetics and larger crystals is consistent with work by Cuthbert and coworkers, where *S. pasteurii* biofilms were cultured with either high (13 g/L) or low (0.13 g/L) nutrient broth concentration^28^. The biofilms produced in low nutrient conditions were sparser and consequently had slower initial ureolysis and calcite mineralization. Calcite crystals were larger when produced in the low-nutrient conditions, which was explained by lower initial saturation state. It is expected from classical nucleation theory that the driving force for nucleation is increased with saturation state, and therefore that the stable crystal nucleus size decreases with increased saturation state^29^. While we did not measure saturation state, our measurements of urease activity and solution calcium are consistent with the interpretation that differences in ureolytic activity, modulated in our study via genetic engineering, alter the solution saturation state and therefore mineralization kinetics and crystal size. The size difference of biogenic calcite precipitates may be of specific importance for some engineering applications. Mitchell and coworkers, for example, found that larger calcite crystals exhibit decreased solubility and may therefore be more useful for co-precipitating metals or radionuclides^13^.

We were able to observe that calcium phosphate phases have a role in both nucleation and growth of CaCO_3_ precipitated by *S. pasteurii* and both engineered *E. coli* microorganisms. Brushite and apatite nanocrystalline sheets were observed by XRD and SEM throughout the 7d experiment in control conditions, as well as for the low-urease engineered *E. coli* strain before the onset of substantial calcite precipitation. Brushite and apatite were also observed concomitant with calcite nucleation in *S. pasteurii* and the high-urease activity engineered *E. coli* strain. Our results from XRD and SEM are in agreement with changes to solution P throughout the 7d experiment. After an immediate drop in P, some portion of P returned to solution with the onset of calcite nucleation and was available to participate in additional precipitation. The saturation state with respect to calcium phosphate would be expected to change continuously during calcite precipitation. In addition, some CaP phases, such as brushite, have decreased solubility in alkaline conditions^30^. We are not the first to observe that CaP plays a role in the nucleation of biogenic CaCO_3_ crystals. Dupraz *et al*. visualized nanoscale CaP nucleated on *S. pasteurii* before CaCO_3_ deposition^31^. In addition, ureolytic microorganisms harvested from cave environments precipitated CaCO_3_ and CaP in lab cultures^1^. However, novel to this study, we directly observed nucleation of calcite on calcium phosphate, and also demonstrate that P is evident in a ring near the polycrystal structure exterior, as well as within the nucleation center (Fig. 4).

The nanoindentation modulus (E_i_) of biogenic calcite was higher for microorganisms with lower urease activity and slower precipitation kinetics. Specifically, E_i_ was approximately 15% higher for the engineered microorganism with lower urease activity, *E. coli* HB101/ure-integration, compared with *E. coli* HB101/pBU11 with higher urease activity. While both microorganisms produced biogenic CaCO_3_ with a range of moduli, *E. coli* HB101/ure-integration produced 63% more crystals with moduli of at least 40 GPa than the engineered *E. coli* strain with higher urease activity. *S. pasteurii*, which has urease activity between that of the two engineered strains, produced calcite with moduli that were between and not significantly different from either engineered *E. coli* strain. The nanoindentation moduli observed in this study were lower than for inorganic CaCO_3_ (~60–80 GPa)^20^. However, nanoindentation moduli of biogenic CaCO_3_ demonstrate a wide range of values; crystals precipitated in lab cultures using non-engineered microorganisms had nanoindentation moduli from 36–65 GPa^21^, while crystals precipitated in the presence of carbonic anhydrase isolated from the sponge *Sycon raphanus* had nanoindentation moduli of 43–47 GPa^32^. On the other hand, cave speleotherms produced via bacterial precipitation have much stiffer crystals, with nanoindentation moduli from 64–126 GPa^33^.

We sought to understand if differences in E_i_ for biogenic CaCO_3_ produced by engineered *E. coli* with low-high urease activity could be attributed to variation in CaCO_3_ polymorphs, crystal porosity, crystal perfection, or inclusion of CaP phases. Differences in nanoindentation modulus and hardness for biogenic CaCO_3_ have been previously attributed to the presence of metastable vaterite or aragonite, in both lab-grown cultures as well as cave speleotherms^19,21,33^. While variation in CaCO_3_ nanomechanical properties may be desirable for engineered applications such as functionally-graded composites and films, achieving this variation via the inclusion of metastable CaCO_3_ polymorphs such as vaterite is not ideal. In our study, only calcite (*i.e*., no vaterite or aragonite) was detected using XRD for any of the 7d samples tested by nanoindentation. Site-matched Raman spectroscopy revealed that every crystal measured with nanoindentation was only calcite and did not include another CaCO_3_ polymorph. Porosity could be altered through differences in crystal nucleation and growth modulated by changing microorganism urease activity, but all indents with significant compaction events (indicating pore collapse) were excluded from analysis. Differences in porosity at the nanoscale cannot be excluded but were not specifically interrogated by this study. Crystal perfection, as measured through crystallinity and also the A280/A712 ratio, also did not significantly correlate to E_i_ for any microorganism. However, from Raman spectra obtained at the same sites as indentation, we detected a significant, negative linear relationship between the presence of CaP phases (brushite and apatite) and E_i_. Both *E. coli* HB101/ure-integration and *S. pasteurii* had similar mean CaP, yet quantity of CaP did not predict E_i_. The specific mechanism by which calcium phosphate influences the nanomechanics of calcite crystals precipitated by the high-urease microorganism is not clear. Calcium phosphates measured by nanoindentation have a wide range of mechanical properties, depending on phase, crystallization, and organic content. Amorphous hydroxyapatite printed on a titanium substrate had a nanoindentation modulus of 65 GPa, which increased to 127 GPa after annealing^34^. Yet lab-grown hydroxyapatite-brushite coatings can have moduli as low as 3–15 GPa^35^. Brachiopods shells containing calcium phosphate demonstrate nanoindentation moduli from 5–50 GPa, with lower values associated with more organic content^20^. In addition, calcium phosphate could, in theory, alter calcite crystal mechanics through assembling at grain boundaries, increasing porosity of the polycrystalline assembly, altering calcite orientation, or potentially myriad other mechanisms. Future investigations varying the phosphate concentration in solution would facilitate investigation of the likely complex role of phosphate on cell growth as well as crystal nucleation, growth, and resulting mechanical properties.

While nanoindentation modulus varied with the kinetics of the precipitating microorganism, hardness (H) did not. Neither H, which was determined from E_i_ and nanoindentation hardness (H_i_) using assumptions of elastic-plastic deformation, nor H_i_/E_i_ significantly differed with microorganism. The ratio of H_i_/E_i_, sometimes referred to as “resilience”, is related to the elastic strain to yield^36^ and is generally higher for biogenic materials when toughness is advantageous for the organism^20,36^. For all biogenic calcites in this study, H_i_/E_i_ values were generally quite high (~0.07), and were equal or greater to those collected from other biogenic materials (e.g., brachiopods and otoliths)^19,20^ and much greater than for inorganic CaCO_3_ (~0.03–0.04), which has relatively lower hardness and higher modulus^20^. Of key importance, we observed that the modulus of biogenic calcite can be selected for higher or lower values through selection of microorganism with appropriate kinetics, while the material hardness is not compromised.

There were several important limitations to this study. First, while we did note that the presence of CaP reduces nanoindentation modulus for crystals precipitated by *E. coli* HB101/pBU11, we did not determine the specific mechanism by which it does so. Second, while engineered *E. coli* improves control of the microorganism and its crystal precipitates compared with a native ureolytic microorganism, these engineered strains are not readily field-deployable, while *S. pasteurii* has already been used successfully in field-scale applications^7^. It is possible that increases to calcite moduli may also be accomplished with *S. pasteurii via* environmental conditions that slow reaction kinetics (*e.g*., lower temperature or nutrient concentration). However, for applications where a high level of control over the microorganism is required (e.g., hybrid living building materials, functionally-graded composites and films), engineered *E. coli* may facilitate the desired level of tailorability over crystal morphology and material properties.

## Conclusions

In this study, we performed physical, chemical, and microscale mechanical assessments of biogenic CaCO_3_ crystals deposited by native ureolytic (*S. pasteurii*) and engineered (*E. coli*) ureolytic microorganisms. Our key findings were that calcite crystal size and nanoindentation moduli could be modulated through metabolically engineering *E. coli* strains to have different urease activities and precipitation kinetics. Crucially, hardness was not changed amongst microorganisms, demonstrating that a choice of higher or lower modulus for biogenic calcite can be made without compromising the resistance of the crystal to irreversible deformation. We also identified that CaP phases have a role in the nucleation and growth of biogenic calcite precipitated by engineered and native ureolytic species. Furthermore, increased CaP was negatively correlated with nanoindentation modulus for the high-urease activity engineered *E. coli* strain (*E. coli* HB101/pBU11). Taken together, the results from this study demonstrate, for the first time, that the morphology and material properties of biogenic CaCO_3_ can be tailored with engineered ureolytic microorganisms—a finding that enables new possibilities for bacteria-mediated design of advanced functional materials.

## Materials and Methods

### Microorganisms and culture conditions

Three microorganisms were cultured for MICP experiments, including *S. pasteurii* ATCC 11859 and two engineered *E. coli* strains: *E. coli* HB101/ure-integration and *E. coli* HB101/pBU11. Pre-culture growth conditions for *S. pasteurii*, at 30 °C, were consistent with previous description^37^. Details of the construction of the engineered *E. coli* strains and pre-culture growth conditions are described in Liang *et al*.^24^. In summary, for the *E. coli* HB101/ure-integration strain, a single copy of the urease gene cluster from *S. pasteurii* was inserted into the genome using a CRISPR-based technique. *E. coli* HB101/pBU11 is a plasmid strain constructed with medium copy number (15–20 copies) of the urease gene cluster. Both *E. coli* strains were pre-cultured at 37 °C in lysogeny broth (LB) containing 50 µM NiCl_2_, which is necessary for ureolysis by *E. coli*^12^. Media for *E. coli* HB101/pBU11 also included ampicillin (100 µg/mL) for plasmid maintenance.

For the experiments, pre-cultured microorganisms were added (at OD_600_ = 0.2) to sterile filtered urea-CaCl_2_ media (20 g/L urea, 10 g/L NH_4_Cl, 3 g/L Difco nutrient broth, 25 mmol NaHCO_3_, 25 mmol CaCl_2_). This media was consistent with media utilized in previous *S. pasteurii* biomineralization experiments^11^ except NiCl_2_ (5 µM) was also included in *E. coli* cultures. Cultures were prepared in 50 mL sterile plastic conical vials and incubated in a shaker at 20 °C, which is a temperature that represents expected conditions for engineering applications. Cultures were sacrificially sampled at four timepoints: 12h, 1d, 2d, and 7d. At the first three timepoints, three replicate cultures per microorganism were sampled. At the 7d timepoint, four replicates per microorganism were sampled to improve statistical power for nanoindentation measurements. Controls included (1) wild-type *E. coli* HB101 with no genetic modifications (Wild-Type control) in urea-CaCl_2_ media and (2) urea-CaCl_2_ media with no microorganisms (Media control). Controls were sampled in triplicate at 1d, 2d, and 7d.

### Solution chemistry and reaction kinetics

At each time point, 10 µL of culture was taken to measure colony forming units (CFUs). This assessment was performed in triplicate. The remaining volume of the 50 mL solution was then vacuum filtered, using 0.22 µm nylon filter paper (Sigma-Aldrich). Precipitates collected from this filtration were used to assess crystal morphology and phase (Section 2.3). From the filtrate, pH was measured on a calibrated Beckman-Coulter electrode. The filtrate was then adjusted with HNO_3_ to pH 1.5 in order to stabilize solution chemistry for inductively coupled optical emission spectroscopy (ICP-OES) measurement of soluble Ca and P. These samples were further prepared using a modified version of the technique developed by Farrell, Matthes, and Mackie^38^. Briefly, 5 mL of a 7:3 mixture of hydrochloric acid and hydrofluoric acid and then 2 mL of nitric acid were added to digestion tubes and heated to 95 °C in a digestion block (HotBlock by Environmental Express) for approximately 2 hours. Samples were then cooled via water bath and brought up to 50 mL with a 1.5% by weight boric acid solution. The samples were then reheated to 95 °C for about 15 minutes and cooled before analysis. Samples were analyzed with a Perkin Elmer SCIEX ICP-OES, model # Elan DRC-e.

The solution depletion rates of Ca (dCa/dt) and P (dP/dt) were evaluated by dividing the difference in elemental concentration between two timepoints by the elapsed time. Because cultures were sacrificially sampled at each timepoint (i.e., different cultures at 12h, 1d, 2d, 7d), the average Ca and P value amongst replicates was used for depletion rate assessment. Depletion rates were calculated at all timepoints for ureolytic microorganisms and controls.

Urease activity was assessed for additional cultures prepared identically to those described in Section 2.1. Briefly, 5 mL of unfiltered solution for each ureolytic microorganism was collected at 1d, 2d, and 7d. These samples were centrifuged and sonicated, and the extract collected for urease activity analysis (Urease Activity Assay Kit, Sigma-Aldrich 357 MAK120).

### Mineralogical assessment of crystal morphology and phase

Precipitates collected from filtration were dried overnight at room temperature in a hood before analysis. These precipitates were divided into samples for scanning electron microscopy (SEM), SEM-energy dispersive spectroscopy (SEM-EDS), and XRD analyses. A portion of the 7d precipitates was also allocated for nanoindentation and site-matched Raman spectroscopy analyses. For SEM characterization of crystal morphology, precipitates were mounted on carbon tape and sputter-coated with a 10 nm coating of platinum. Crystal morphology was evaluated with a Hitachi SU3500 set at an accelerating voltage of 5 kV, working distance of 8 mm, and spot size of 30. Crystals were visualized at 500x and 1500x for each sample. Crystal area was evaluated at 7d for each replicate using 500x images and ImageJ. All in-focus and well-defined crystals were measured for each image. For SEM-EDS, samples were sputter-coated with gold. Composition of crystals was assessed with a JEOL 6480 at an accelerating voltage of 15 kV, working distance of 10 mm, and spot size of 50. For XRD, dried precipitates were rinsed in DI water and dried on no-background silicon disks for fingerprint XRD. A Siemens D500 X-ray diffractometer analyzed samples from 5 to 65° 2θ using CuKαX-ray radiation with a stepsize of 0.02° and a dwell time of 2 s per step. Crystal phases were identified using Jade software (MDI, version 9) and the International Centre for Diffraction Data (ICDD) 2003 database.

Samples for nanoindentation and Raman spectroscopy were prepared by embedding precipitates in Epoxy (EpoxiCure 2, Buehler). Embedded disks were then ground with 600 and 1200 grit papers and polished with progressively fine diamond-oil suspension (9, 6, 3, 1, 0.025 µm grit) (Buehler). A polished disk was prepared for each of the four replicates per microorganism. After nanoindentation and Raman spectroscopy (described below), embedded crystals were assessed for elemental composition using wavelength-dispersive spectroscopy (WDS). The polished disks were first sputter-coated with 20 nm of carbon. Elemental maps for Ca and P were obtained for several crystals for each microorganism using a JEOL-8230, using 30 nA, 50 ms dwell, 2 µm spot size and collecting over 512 × 512 pixels. The elemental ratio of P:Ca was calculated for all WDS maps.

### Site-matched microscale assessment of CaCO_3_ phase, composition, and mechanical properties

Raman spectroscopy (Renishaw inVia) was first employed to identify CaCO_3_ crystal phase and the presence of calcium phosphate impurities for a sample of crystals within each polished disk. This microscale compositional characterization was followed by site-matched nanoindentation (Hysitron TI950) enabled through use of a custom stage. Raman spectra (785 nm excitation, 50x objective) first confirmed the crystal polymorph. The baseline for each acquired Raman spectra was subtracted using an 11th order polynomial fit (Renishaw WIRE, v4.4). Peak areas for CaCO_3_ lattice and *v*_4_ modes areas were assessed for calcite (lattice: 280, *v*_4_: 712), aragonite (lattice: 210, *v*_4_: 701–705 cm^−1^), and vaterite (lattice: 300, *v*_4_: 750 cm^−1^)^23,39^. The ratio of the lattice to *v*_4_ calcite peaks (A280/A712), shown in a prior study to positively correlate with crystal size, was also calculated^40^. The full width at half-max intensity (FWHM) of *v*_1_ carbonate (~1085 for all CaCO_3_ polymorphs) was calculated as a measure of crystal perfection; wider peaks are associated with poorly crystalline material^39^. The *v*_1_ phosphate peak is known to have distinct positions for brushite (986 cm^−1^), apatite (960 cm^−1^), and carbonated apatite (970 cm^−1^)^41^. These peaks areas were assessed, and ratios of brushite:*v*_1_ carbonate, apatite:*v*_1_ carbonate, and carbonated apatite:*v*_1_ carbonate were calculated. All Raman spectra were analyzed using a custom MATLAB (v2017b) script.

Following Raman spectroscopy, nanoindentation was performed with a Berkovich tip in displacement-control mode to a maximum displacement of 500 nm (Hysitron TI950). The load function was 10 s ramp, 10 s hold, 10 s unload. Indents were placed a minimum of 10 µm away from any sample edges and previous indents. Indents were excluded from analysis if load-displacement curves showed evidence of a compaction event or poor surface contact (Supplementary Information Fig. 1). A total of 15–20 indents with corresponding Raman spectra were analyzed for each of the four disks per microorganism, with a total of 68–69 locations assessed for each microorganism.

Load-displacement curves were analyzed using the Oliver-Pharr approach^42^ and custom MATLAB script. Briefly, stiffness (S) was determined from the derivative of the initial portion of the unloading curve with the assumption of elastic behavior. The tip contact area (A_c_) is calibrated as a function of contact depth (h_c_). The reduced modulus (E_r_) was calculated according to:

1 $${E}_{r}=\frac{\sqrt{\pi }S}{2\sqrt{{A}_{c}{h}_{c}}}$$

The reduced modulus is a function of sample (s) and tip (t) modulus (E_s_, E_t_) as well as Poisson’s ratios of the sample (*v*_s_) and tip (*v*_t_):

2 $$\frac{1}{{E}_{r}}=\frac{(1-{v}_{s}^{2})}{{E}_{s}}-\frac{(1-{v}_{t}^{2})}{{E}_{t}}$$

If tip properties are known (here, E_t_ = 1140 GPa, *v*_t_ = 0.07) and if the sample Poisson’s ratio is known, the sample modulus can be determined. Here, the Poisson’s ratio of biogenic CaCO_3_ was not determined. Therefore, we present the indentation modulus (E_i_), in which no assumption of Poisson’s ratio is necessary:

3 $${E}_{i}=\frac{({E}_{s})}{1-{v}_{s}^{2}}={(\frac{1}{{E}_{r}}+\frac{(1-{v}_{t}^{2})}{{E}_{t}})}^{-1}$$

The value of E_i_ quantifies the resistance to elastic (reversible) deformation during indentation. Indentation hardness (H_i_) was also calculated for every indent by measuring the maximum load and the corresponding contact area created by the maximum load:

4 $${H}_{i}=\frac{Pmax}{{A}_{c}}$$

H_i_ necessarily incorporates contributions from both elastic and inelastic deformations. For materials that are not perfectly plastic, such as CaCO_3_, H_i_ is different than hardness (H) assessed by bulk-scale techniques (e.g., Rockwell testing)^36^. However, assuming elastic-plastic material deformation—an assumption that is not valid for substantially viscoelastic materials—a constitutive relationship exists between hardness, H, and H_i_ and E_i_^36^:

5 $$H=\frac{{H}_{i}}{{(1-\sqrt{{H}_{i}/{E}_{i}}\sqrt{2/\tan (\beta )})}^{2}}$$

The resulting value of H is independent of E_i_. The value β is the equivalent cone angle of the indenter and equals 70.32° for a Berkovich tip. For each nanoindentation curve, E_i_, H_i_, and H were calculated. The ratio of H_i_/E_i_ was also calculated, which is interpreted as the relative resistance to inelastic deformation in the contact zone during nanoindentation^20,36^.

### Statistical methods

Measurements from nanoindentation (E_i_, H_i_, H, H_i_/E_i_) as well as morphology (crystal area) were averaged for each replicate for each microorganism. Nanoindentation measures were compared between microorganisms (*S. pasteurii*, *E. coli* HB101/ure-integration, *E. coli* HB101/pBU11) using one-factor ANOVA. Post-hoc testing was performed using a Tukey correction to adjust critical alpha for family-wise error. The definition of significance was set *a priori* to p < 0.05. For all models, residuals were checked for normality and homoscedasticity. The Pearson product-moment correlation was performed to assess the strength of the relationship between Raman spectroscopy measures of crystal size and composition, as well as crystal nanoindentation modulus. All analyses were performed using Minitab (v18).

## Supplementary information

Supplementary Information
