# Supplementary material for: Engineered Ureolytic Microorganisms Can Tailor the Morphology and Nanomechanical Properties of Microbial-Precipitated Calcium Carbonate
Source: Sci Rep. 2019 Oct 11;9:14721. doi: 10.1038/s41598-019-51133-9 (PMC6789151; doi:10.1038/s41598-019-51133-9)
Supplement: Supplementary file 1 — Supplementary Information [file 41598_2019_51133_MOESM1_ESM.docx]

**Supplementary Information**

Engineered Ureolytic Microorganisms Can Tailor the Morphology and Nanomechanical Properties of Microbial-Precipitated Calcium Carbonate

Chelsea M. Heveran, Liya Liang, Aparna Nagarajan, Mija H. Hubler, Ryan Gill, Jeffrey C. Cameron, Sherri M. Cook & Wil V. Srubar III

**Supplementary Figure 1: Assessment of nanomechanical properties, phase, and composition of biogenic CaCO_3_.** (A) SPM image of nanoindentation indent, showing that neither pile-up nor fracture occurred upon indentation. (B) Nanoindentation load-displacement curves for the three ureolytic microorganisms. (C) Representative Raman spectra for biogenic calcite.
